# Faceting/Roughening of WC/Binder Interfaces in Cemented Carbides: A Review

**DOI:** 10.3390/ma16103696

**Published:** 2023-05-12

**Authors:** Boris B. Straumal, Igor Konyashin

**Affiliations:** 1 Chernogolovka Scientific Center, Osipyan Institute of Solid State Physics, Russian Academy of Sciences, Ac. Osipyan Str. 2, 142432 Chernogolovka, Russia; 2 Element Six GmbH, Staedeweg 18-24, 36151 Burghaun, Germany; igor.konyashin@e6.com

**Keywords:** cemented carbides, equilibrium shape, faceting, roughening, grain boundaries, interphase boundaries

## Abstract

Hardmetals (or cemented carbides) were invented a hundred years ago and became one of the most important materials in engineering. The unique conjunction of fracture toughness, abrasion resistance and hardness makes WC-Co cemented carbides irreplaceable for numerous applications. As a rule, the WC crystallites in the sintered WC-Co hardmetals are perfectly faceted and possess a truncated trigonal prism shape. However, the so-called faceting–roughening phase transition can force the flat (faceted) surfaces or interfaces to become curved. In this review, we analyze how different factors can influence the (faceted) shape of WC crystallites in the cemented carbides. Among these factors are the modification of fabrication parameters of usual WC-Co cemented carbides; alloying of conventional cobalt binder using various metals; alloying of cobalt binder using nitrides, borides, carbides, silicides, oxides; and substitution of cobalt with other binders, including high entropy alloys (HEAs). The faceting–roughening phase transition of WC/binder interfaces and its influence on the properties of cemented carbides is also discussed. In particular, the increase in the hardness and fracture toughness of cemented carbides correlates with transition of WC crystallites from a faceted to a rounded shape.

## 1. Introduction

Hardmetals (or cemented carbides) were invented in 1923 and later became one of the most important materials used in engineering. Typically, cemented carbides consist of brittle WC grains surrounded by a ductile, usually cobalt-based metallic binder. The WC-Co composites have high strength of above 3000 MPa (for 10 wt.% Co) [1], high density (≈15 g/cm^3^ [1,2]), excellent hardness (up to 20 GPa [1]) and fracture toughness (>10 MPa·m^1/2^ depending on a WC grain size [3]). This unique combination of fracture toughness, abrasion resistance and hardness makes WC-Co cemented carbides irreplaceable for numerous applications [4,5,6].

**Figure 1:**
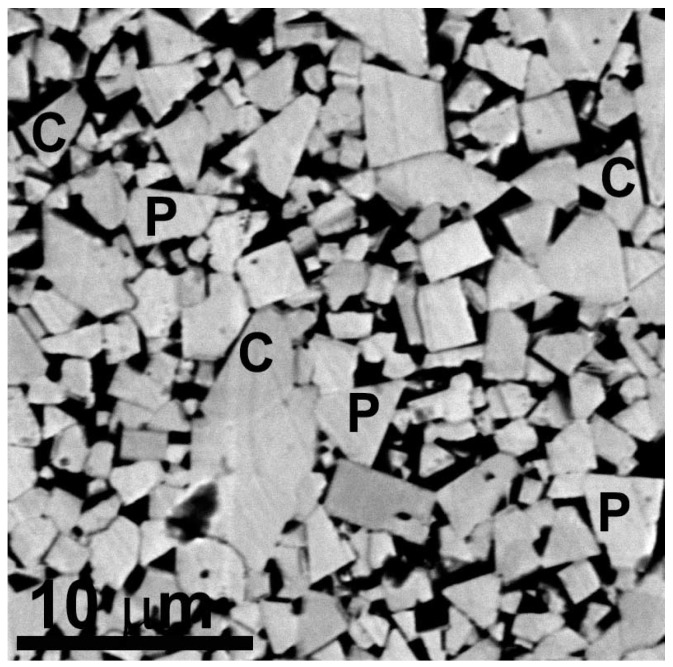
SEM micrograph of WC/Co cemented carbide with ultra-coarse WC crystallites manufactured via liquid-phase sintering at 1380 °C. Co-binder appears black in this micrograph and WC crystallites appear white. Some WC/WC grain boundaries (GBs) can be completely wetted via Co-based melt (marked with letters C) and have zero contact angle with liquid phase. Other WC/WC GBs are incompletely (or partially, marked with letters P) wetted via melt. They have a non-zero contact angle and are marked with letters C in micrograph. Reprinted with permission from authors of [4]. Copyright 2017 Elsevier.

**Figure 2:**
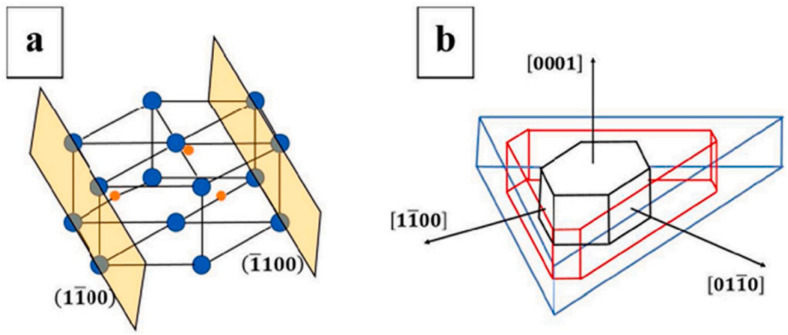
(**a**) is hexagonal close-packed (hcp) lattice tungsten carbide, while (**b**) is equilibrium shape of tungsten carbide. Reprinted with permission from authors of [6]. Copyright 2020 Elsevier.

As a rule, the WC grains in the sintered WC-Co hardmetals are perfectly faceted and possess a truncated trigonal prism shape (see Figure 1 and Figure 2 and [4]). Nevertheless, the so-called faceting–roughening phase transition can force the flat (faceted) surfaces or interfaces to become curved [7,8]. The transition from flat to curved (rough) surfaces or interfaces can be proceeded through the modification of their misorientation or inclination [9,10], a change in temperature [11,12] or alloy composition [13].

**Figure 3:**
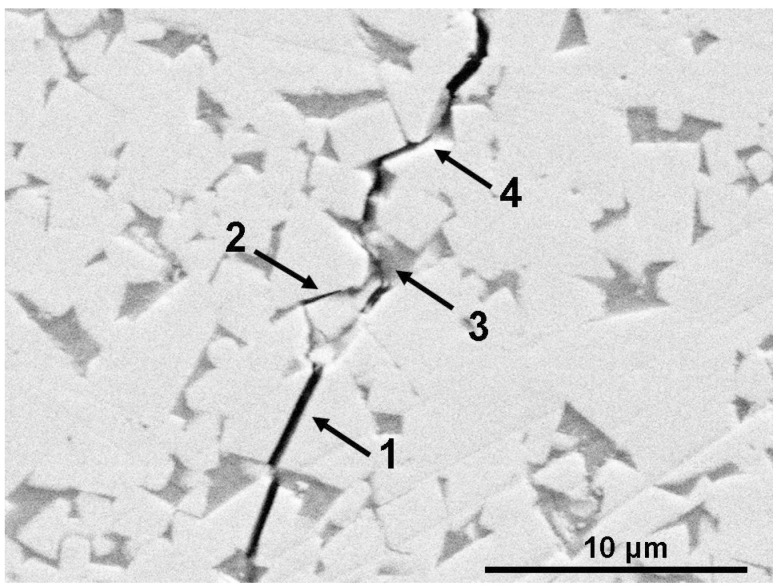
SEM micrograph of WC/Co cemented carbide with ultra-coarse WC crystallites manufactured via liquid-phase sintering at 1380 °C. Co-binder appears dark-grey in this micrograph and WC crystallites appear white. Crack is produced using Vickers indentor and appears black. Digits and arrows show crack permeating WC grains (1), moving between WC grains (2), moving through or between Co grains (3) or moving along WC/Co interfaces (4).

We can see that these facts concerning faceting–roughening of interfaces [7,8,9,10,11,12,13] were observed in molybdenum [7,8], copper [9,11,13], aluminum [10] or zinc [12]. Nevertheless, they are of a very general nature and can be applied to other classes of materials. Since the WC grains are usually well faceted (Figure 1, Figure 2 and Figure 3), we can expect that different factors (such as temperature, pressure, composition, chemical or mechanical treatment) could modify the ideal faceted shape of WC crystallites observed in conventional WC-Co cemented carbides. Moreover, we can expect that the transition from a faceted to a rough shape among WC/Co interfaces could, in turn, modify the properties of WC-Co cemented carbides. For example, the penetrating crack in WC-Co cemented carbides can take the following actions: (1) permeate the WC grains, (2) move between the WC grains, (3) move through or between the Co grains or (4) move along the WC/Co interfaces (marked with arrows in Figure 3). Therefore, it is easy to imagine that the trajectory of crack can be controlled using the shape of WC/Co interfaces. In turn, the structure and properties of WC-Co cemented carbides can depend on the faceting–roughening of WC grains. To the best of our knowledge, such inter-relation was never analyzed in past studies. This issue constitutes the definite research gap that should be closed.

Closing this gap is the goal of this survey paper. Here, we will analyze how various factors can influence the faceted shape of WC grains in the cemented carbides. Relevant factors include modification of fabrication parameters of conventional WC-Co cemented carbides (Section 2); low alloying of conventional cobalt binder using various metals (Section 3); alloying of cobalt binder using nitrides, borides, carbides, silicides, oxides (Section 4); and substitution of cobalt with other binders (Section 5), including high entropy alloys (HEAs) (Section 6). We will also discuss how the faceting–roughening of WC/binder interphase boundaries can modify the properties of cemented carbides.

## 2. Modification of Fabrication Parameters of Conventional WC-Co Cemented Carbides

Wang et al. prepared the WC-12 wt.% Co cemented carbides using the selective electron beam melting (SEBM) [14]. Afterwards, the samples were sintered at 1400 °C under argon at 5 MPa. The microhardness obtained after the sintering stage (1686 HV-Vickers units) was lower than that of the SEBM-fabricated sample (1929 HV). Vise versa, the compressive strength after the sintering (1839 MPa) was higher than immediately after SEBM (1770 MPa). The average friction coefficient of as-fabricated samples was 0.548, with a wear rate of 0.0055 mm^3^. The sintering at 1400 °C increased these values by 59.7% and 142%, respectively.

**Figure 4:**
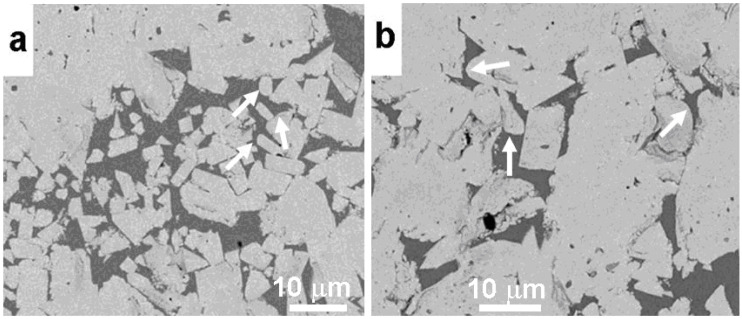
SEM micrographs of WC-12 wt.% Co cemented carbide fabricated via SEBM before (**a**) and after (**b**) heat treatment. White arrows show the rounded corners of WC crystallites. Reprinted with permission from authors of [14]. Copyright 2022 Elsevier.

In the micrographs shown in Figure 4, it is well visible that many WC crystallites in the WC-12 wt.% Co cemented carbide fabricated using SEBM had rounded corners [14]. They are shown with white arrows (Figure 4a). Such rounded corners were absent in the WC-Co cemented carbides sintered in a conventional process (Figure 1). Figure 4b shows that after additional sintering at 1400 °C, the rounded corners of WC crystallites were still present.

The original shape of WC single crystals after the carburization stage followed by gentle de-agglomeration was rounded; thus, if such WC single crystals are only mixed with Co and not subjected to milling leading to the formation of fine-grain WC fraction, the WC grains remain rounded in the microstructure after sintering. Cemented carbides with rounded carbide grains could be obtained via cladding coarse-grain WC powders with cobalt [15]. This made it possible to eliminate the formation of a fine WC fraction during milling of WC-Co graded powders. As a result, the recrystallization of such a fine-grain fraction of large WC grains during sintering could be suppressed, thus leading to the elimination of the process of faceting such large grains of tungsten carbide during sintering.

Thus, in general, the roughening of the WC/binder interfaces can be obtained either through eliminating the process of the fine-grain fraction formation during milling or, if such a fine-grain WC fraction forms, suppressing the process of its dissolution in a liquid binder, followed by its recrystallization and the growth of large facetted WC grains during sintering.

The changes in the shape of WC grains were also observed through the preparation of conventional WC-Co cemented carbides using laser powder bed fusion [16], hot isostatic pressing [17] and spark plasma sintering [18], as well as through varying the carbon [19] and cobalt content [20].

## 3. Alloying Conventional Cobalt Binders Using Various Metals

In [21], the conventional cobalt binder was alloyed with Al, Ni and W. The four alloys were prepared, i.e., Co-12 at.% Al-11 at.% W, Co-15 at.% Ni-12 at.% Al-11 at.% W, Co-30 at.% Ni-12 at.% Al-11 at.% W and Co-45 at.% Ni-12 at.% Al-11 at.% W. Thus, the Ni-to-Al atomic ratio varied (it was 0, 1.25, 2.5 and 3.75), while the Al-to-W atomic ratio was constant at 1.1. For synthesis, the elementary Co, Al, Ni and W powders used were of powder metallurgical standard grade. After infiltration, all samples were annealed for 48 h at 950 °C in argon atmosphere. Thus, the Ni-based superalloy was used as a binder. It was demonstrated that different γ/γ’ microstructures in binder of cemented carbides could be produced through different interactions between Al, Ni, W and C in the binder alloy. If the binders had no nickel, they also had no γ’ precipitates. The micrographs in [21] show that the shape of WC crystallites differs from truncated prism shown in Figure 1. It looks as if new facets appeared in the equilibrium shape. The corners of WC crystallites became ever-more rounded with increasing Ni content [21].

The addition of ruthenium to the cobalt binder can also modify the shape of WC grains. In [22,23], 0 to 6 wt.% Ru was added to the conventional WC-10 wt.% Co cemented carbides. When the ruthenium concentration increased, the fracture toughness, density, hardness and coercivity of the composites changed. When the ruthenium concentration reached 4%, the hardness increased from 1290 to 1330 (HV30), and the fracture toughness grew from 16.8 to 24.2 (MPa·m^1/2^), compared with the alloy without ruthenium. The analysis of microstructures of Ru-alloyed WC-10 wt.% Co cemented carbides (Figure 1 from [22]) shows that the addition of ruthenium modified the shape of WC crystallites, which became slightly rounded. This effect was maximal at 4% Ru and correlated, therefore, with maximal fracture toughness and hardness (see Table 1).

**Figure 5:**
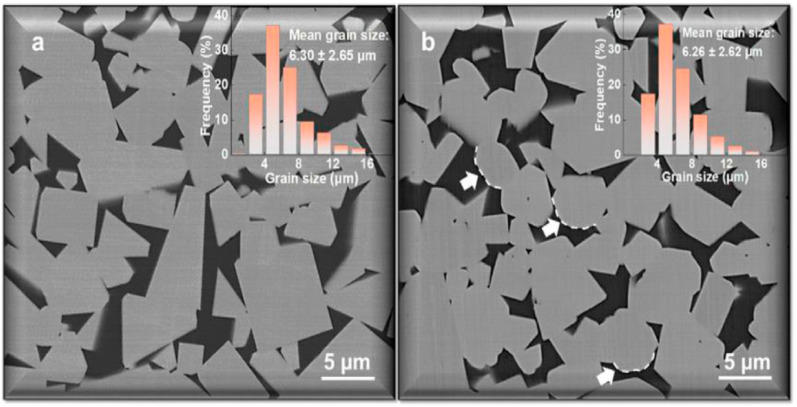
SEM micrographs obtained in BSE mode of as-sintered ultra-coarse grained cemented carbides (**a**) WC-8Co and (**b**) WC-8Co-0.8TaC. Rounded edges of WC crystallites are marked using bright arrows. Insets show the corresponding distributions of WC grain sizes. Reprinted with permission from authors of [26]. Copyright 2022 Elsevier.

Hu et al. proposed to improve the high-temperature compression strength through adding activated TaC nanoparticles to ultra-coarse cemented carbides [26]. This effect was achieved through modulating the Co matrix using TaC nanoparticles, as well as through solid-solution strengthening using Ta atoms. The milled WC, Co and TaC powders were sintered for 80 min at 1480 °C in argon atmosphere at 60 bar. The addition of 0.8 wt.% TaC to the WC-8 wt.% Co indeed led to about a 20% increase in the compressive strength of in the temperature interval 600–1000 °C. The addition of tantalum also led to the rounding of the corners of WC crystallites (see Figure 5). The rounded edges of WC grains are marked in Figure 5 with the white arrows.

**Figure 6:**
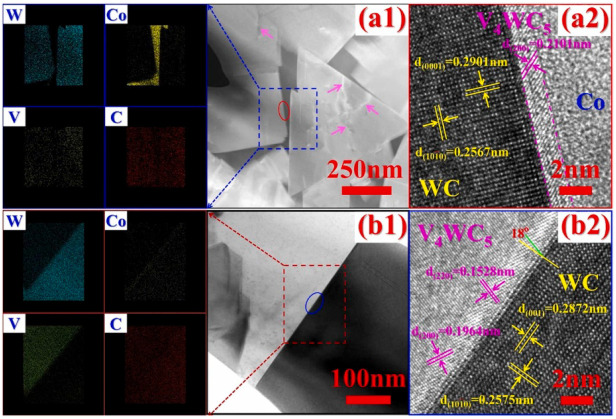
TEM and HRTEM micrographs and elemental distribution for C_3.5_V_2_ (WC-12 wt.% Co-2 wt.% VC) and C_3.5_V_6_ (WC-12 wt.% Co-6 wt.% VC) alloys after sintering for 2 h at 1400 °C: (**a**) C_3.5_V_2_; (**a2**) is the HRTEM micrograph of the red oval area marked in (**a1**). (**b**) C_3.5_V_6_. (**b2**) is the HRTEM micrograph of the blue oval area marked in (**b1**). Reprinted with permission from authors of [27]. Copyright 2022 Elsevier.

**Figure 7:**
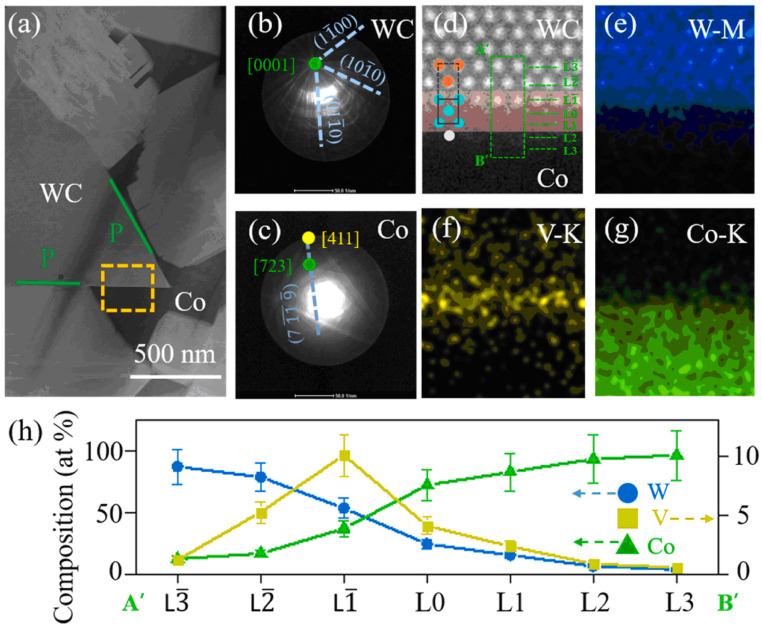
(**a**) TEM HAADF micrograph of WC/Co interphase boundary. Yellow box shows contour of respective FIB lamella. (1,0,−1,0) plane terminates WC/Co interphase boundary on WC side. At a zero-tilt condition, we acquired the Kikuchi diffraction patterns of (**b**) WC grain and (**c**) surrounding cobalt binder. (**d**) WC/Co interphase boundary viewed along a [0001] zone axis of WC (expanded HAADF image). (**e**–**g**) show atomic-resolution EDS mapping. These maps permit us to reveal segregation in this WC(P)/Co interphase boundary. (**h**) shows corresponding concentration profiles of tungsten, vanadium and cobalt across interphase boundary (along A’B’ track in (**d**)). A L-1 layer denotes outermost surface of WC grain. At L0 layer, abrupt transition begins at the interphase boundary. Reprinted with permission from authors of [33]. Copyright 2022 Elsevier.

**Figure 8:**
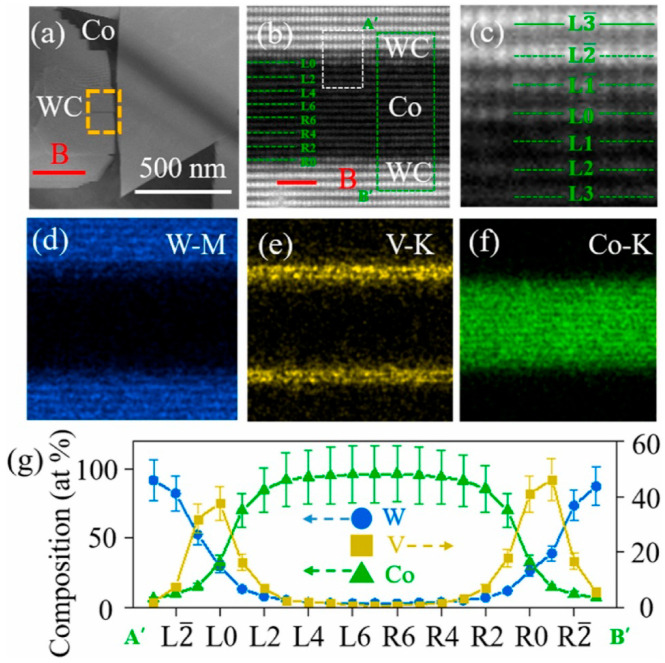
(**a**) TEM HAADF micrograph of two coherent WC/Co interphase boundaries. These two coherent WC/Co interphase boundaries are enclosed usinh (0001) planes on tungsten carbide side. (**b**) contains atomic-resolution HAADF image. (**c**) Shows expanded image of white box marked in (**b**). (**d**–**f**) are EDS concentration maps of tungsten, vanadium and cobalt for interfaces. For these maps, WC grain is setting at a [1,0,−1,0] zone axis. (**g**) Concentration profiles of tungsten, vanadium and cobalt across A’B’ line in (**d**). A’B’ line intersects both interfaces. Reprinted with permission from authors of [33]. Copyright 2022 Elsevier.

The faceting–roughening transitions of surfaces and interfaces, as well as modification of facets after addition of second component, is a well-known outcome for various metallic alloys [8,13,31,32]. This process occurs due to the different adsorption of doping atoms in different facets and rough portions of surfaces and interfaces. In [27], such differences were also observed using the high-resolution transmission electron microscopy (HRTEM) for cemented carbides. The WC-12Co-*x*VC (with *x* = 0, 1, 2, 4, 6, 8 wt.%) cemented carbides were synthesized using an in situ synthesis method. This method had the following steps: precursor formation, deoxidization and carbonization-sintering [27]. The VC addition led to an increase in Vickers hardness (see Table 1). HRTEM permitted us to observe the about 2 nm thin V-enriched adsorption layers in the WC/Co interphase boundaries (Figure 6). The thickness of the adsorption layers slightly increased with an increase in VC concentration from 2 to 6 wt.% (Figure 6). The interfacial vanadium segregation in V-doped WC-Co cemented carbides is facet-dependent [33]. The V-doped WC-Co cemented carbide samples were manufactured via vacuum sintering [33]. The WC (4.0 μm), VC (1.5 μm), and Co (0.8 μm) powders were used. The powders were mixed and ball-milled. The V content was 0.30 wt.%, while that of cobalt was 10 wt.%. The mixed powder was vacuum dried, pressed into pellets at 150 MPa and sintered in vacuum for 1 h at 1450 °C. The aberration-corrected scanning TEM was used to study the segregation of V at WC/Co interphase boundaries and WC/WC grain boundaries in WC-Co cemented carbides doped using vanadium on the atomic level [33]. It was found that the orientation of low-index planes controls the segregation of vanadium. This is particularly true for the basal [(0001) plane, see Figure 2b] and prismatic [(1,−1,0,0) and (0,−1,1,0) planes, see Figure 2b] planes. These facets of WC crystallites prevail in WC-Co composites. Vanadium solute atoms weakly segregated at the WC interfaces were terminated with prismatic (1,−1,0,0) or (0,−1,1,0) planes. The prismatic WC interfaces contained, at the outermost surface, a monolayer with only about 10 at.% V (Figure 7). In contrast, the basal planes of WC grains contained a segregation bilayer with a higher vanadium concentration of about 25 at.% (Figure 8). Moreover, the crystallographic dependence of interfacial segregation is valid for grain and interphase boundaries because grain boundaries frequently possess prismatic or basal facets of tungsten carbide grains [33].

Minor shape changes in WC grains were also observed through the alloying of conventional WC-Co cemented carbides using Fe [34,35], Fe–Ni [28,29,36,37,38,39], Fe–Ni–Cr [40,41], Fe–Ni–Cr_3_C_2_ [42], Re [43], Ni, Nb [44] and Ni–Al–W [45].

## 4. Alloying of Cobalt Binder Using Nitrides, Borides, Carbides, Silicides, Oxides

In [46], the carbothermal reduction–nitridation (CRN) technology was utilized to produce three different quaternary solid-solution powders of (Ti, M)(C_1−x_, N_x_) (M = Ta, Nb, W, *x* = 0.15, 0.20 and 0.26). These powders had various nitrogen contents. The nitrogen concentration in these quaternary carbonitrides strongly influenced the composition, morphology and the grain size of the synthesized cemented carbides. It was also shown that the alloying the carbonitride could reduce the grain growth of WC. The mechanical properties of these hardmetals were improved with an increase in the nitrogen concentration. The analysis of micrographs shows that nitrogen content also influenced the shape of WC grains. At *x* = 0.15, the WC grains became slightly rounded. Through an increase in the nitrogen content towards *x* = 0.20 and 0.26, the sharp corners appear again. It is interesting that transverse rupture strength and hardness follow this non-monotonous behavior of interface faceting (see Table 1).

Agyapong et al. utilized the selective laser melting (SLM) to manufacture the WC-17 wt.% Co-based cemented carbides [47]. These cemented carbides were reinforced with hexagonal boron nitride (10, 5 and 3 vol.% of h-BN). Differently to the unmodified WC-17 wt.% Co samples, none of the reinforced composites experienced the undesirable W_2_C phase. Moreover, the hexagonal BN phases were not found in the samples. The Co–W–B and W–B phases were instead found. The alloying with h-BN drastically changed the shape of WC crystallites. Instead of truncated prisms, they developed a thin flat platelet shape. This transition was especially pronounced in the samples with 5 vol.% of h-BN. The samples with h-BN had better mechanical properties than the undoped WC-17 wt.% Co-based cemented carbides. The samples with 5 vol.% of h-BN possessed the highest fracture toughness of 6.97 MPa·m^−1^. This outcome occurred because the samples with 5 vol.% of h-BN had the high-volume fraction of WC platelets. Such platelets contained few basal planes. These facts resulted in lower concentrations of stacking faults interfering with crack propagation.

Minor changes in the shape of WC crystallites were also observed through the alloying of conventional WC-Co cemented carbides using Ti_3_SiC_2_ [48], WCoB [49], Al_2_O_3_, La_2_O_3_, Y_2_O_3_, CeO_2_ [50], NbC [44], CrC [51], graphene oxide [52], MgO [53], Al_2_O_3_, Y_2_O_3_ [21], Ti(C_0.5_, N_0.5_)-Mo [54], VC, Cr_3_C_2_ [55], multiwalled carbon nanotubes [56,57,58], SiC [59], graphene [60], VC [61], TiC [62,63], VC and (Ta, Nb)C [62]. It appeared to be challenging to maintain carbon nanotube and graphene during sintering, as carbon easily dissolves in both solid and liquid cobalt at elevated temperatures.

## 5. Substitution of Cobalt with Other Binders

**Figure 9:**
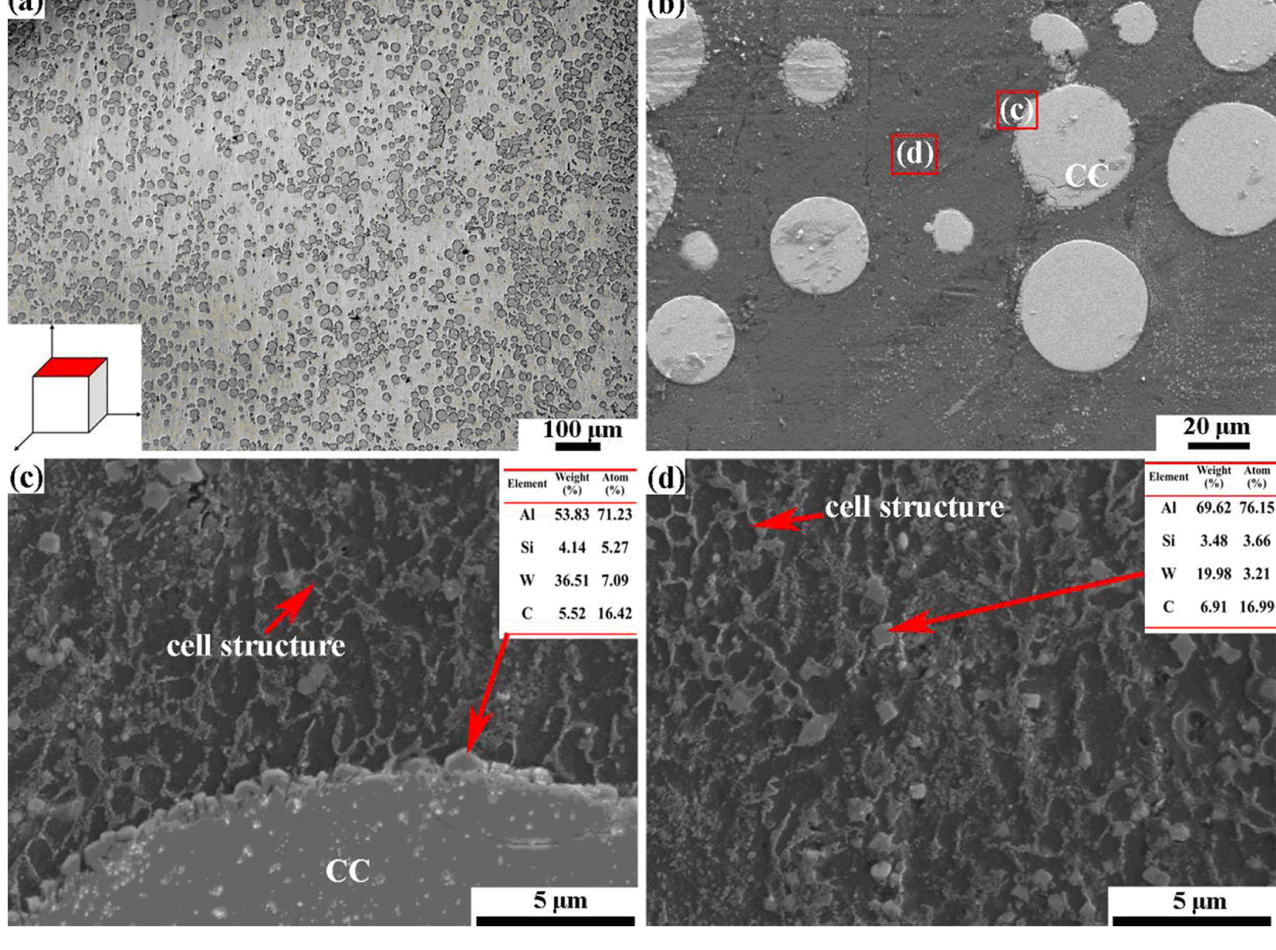
Microstructures of CC-Al-Si-10Mg cemented carbides. (**a**) light micrograph, investigated surface (red), is perpendicular to the laser beam during SLM. (**b**) SEM image. (**c**,**d**) are SEM images and EDX composition data. Respective analysis positions are marked in (**b**) as (**c**,**d**). Reprinted with permission from authors of [64]. Copyright 2022 Elsevier.

**Figure 10:**
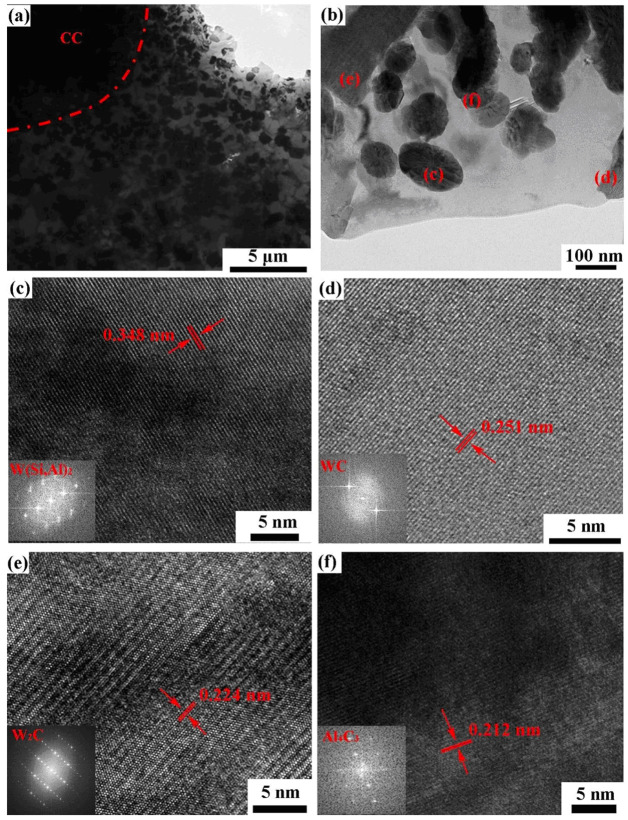
TEM analysis of CC-Al-Si-10Mg cemented carbides; (**a**,**b**) contain the TEM images at low and high magnification, respectively. (**c**–**f**) are high-resolution lattice and FFT images corresponding to positions (**c**–**f**) in (**b**). These images correspond to W(Si, Al)_2_ (**c**), WC (**d**), W_2_C (**e**) and Al_4_C_3_ (**f**) carbides. Reprinted with permission from authors of [64]. Copyright 2022 Elsevier.

The most radical modification of the shape of WC grains can be found in [64]. In this work, the gas-atomized Al–Si–10Mg alloy with mean particle size of 35 μm and cast tungsten carbide (CC, with mean particle size of 42 μm) powders were sintered using the selective laser melting (SLM) process. It is clearly visible in Figure 9 that, in combination with AlSi10Mg binder, the WC grains possessed a spherical shape, which was completely different from truncated trigonal prism shape of conventional cemented carbides with Co binder (Figure 1). The small individual particles of W(Si, Al)_2_, WC, W_2_C and Al_4_C_3_ carbides reprecipitated in the AlSi10Mg melt had an appearance close to the spherical CC particle (see Figure 10), as well as having a rounded and non-faceted shape.

Xu et al. substituted the cobalt binder for the 45Cr-18Ni alloy [25]. The WC–45 wt.% Cr–18 wt.% Ni alloys were manufactured using Binder Jetting, followed by sintering. The manufactured parts had a relatively high density of 98.63%, were crack-free and possessed hardness of >1200 HV and compressive strength of >2200 MPa. They had good corrosion and oxidation resistance, as well as optimal dimensional stability. Therefore, they could be applied as mould material when working at high temperatures. Such moulds require the high corrosion, wear and oxidation resistance. In the microstructure (see Figure 6 in [25]), it was remarkable that not only were the edges of the WC crystallites rounded (as shown in Figure 3), but about a half of WC/binder interphase boundaries were also curved.

**Figure 11:**
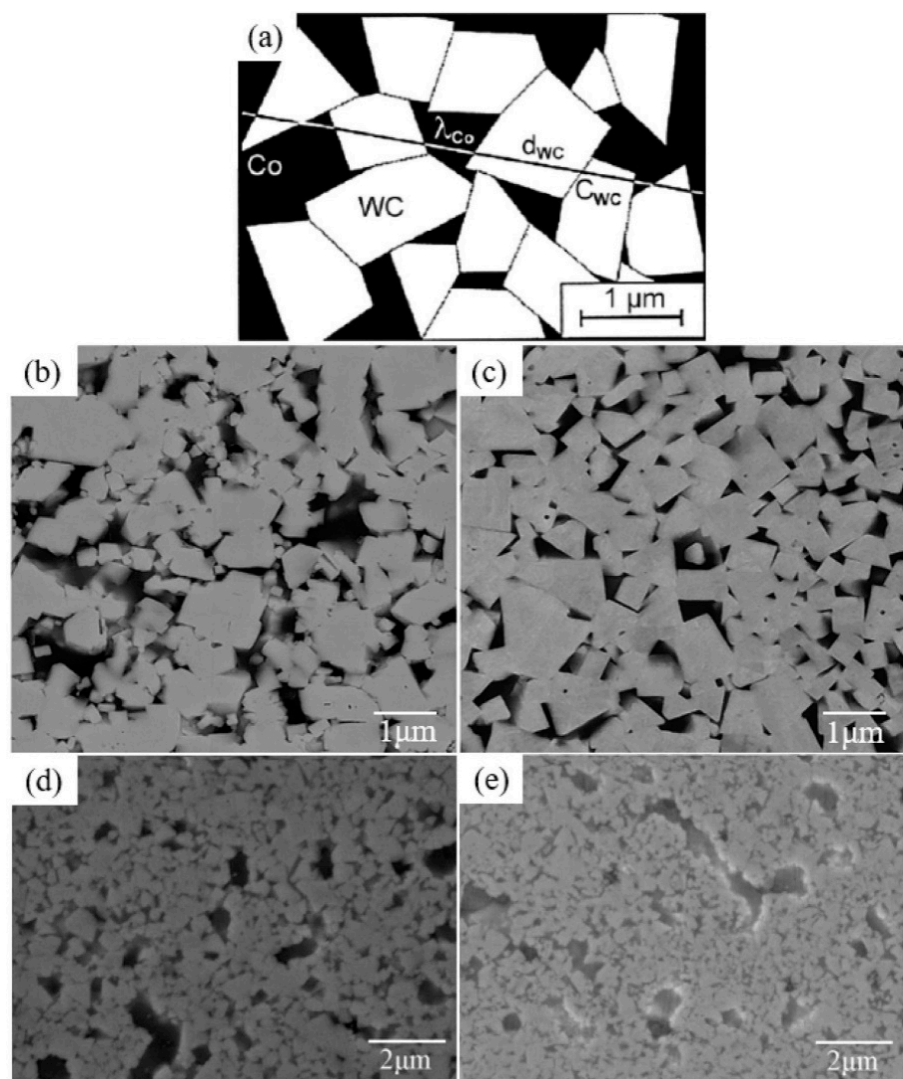
(**a**) Scheme showing the parameters describing microstructure of cemented carbides. SEM micrographs produced with either (**b**) a commercial WC-18 vol.% Co or (**c**) WC-25 vol.% FeAl cemented carbide produced via SPS. SEM micrographs of WC-25 vol.% FeAl produced via LPS with either (**d**) enhanced oxygen content or (**e**) carbide inhibitors. Reprinted with permission from authors of [65]. Copyright 2022 Elsevier.

In [65], the conventional WC-18 vol.% Co cemented carbide (Figure 11b) was compared with composite where the cobalt binder was substituted with FeAl. In Figure 11c, the microstructure is shown for the WC-25 vol.% FeAl cemented carbide produced via solid phase sintering (SPS). In Figure 11b, the edges of the WC grains were even less rounded than in the commercial WC-18 vol.% Co composite manufactured through t liquid phase sintering (LPS). The increased oxygen concentration (Figure 11d) and presence of carbide inhibitors (Figure 11e) during LPS made the WC grains even more rounded.

Minor changes in shape of WC crystallites were also observed through the substitution of cobalt binder in WC-based cemented carbides using Fe [66,67,68], Ni [69,70,71,72,73,74], Al [75,76], Fe–Mn [77,78,79,80,81,82], Fe–Cu [83], Fe–Ni–Mo [84], Ni_3_Al [85,86,87,88,89,90,91,92], Ni–Cr [93], Y_2_O_3_ [94], Al_2_O_3_ [85,95,96], Ni, CoNi, NiCr, CoCr, CoNiCr, NiCrMo [97], MgO [98,99], ZrO_2_ [100,101,102], La_2_O_3_ [103,104], iron aluminides [65,105,106,107,108,109], stainless steel [110,111,112,113,114,115] and titanium aluminides [116,117,118,119].

## 6. Faceting–Roughening of WC/HEA Binder Interfaces

**Figure 12:**
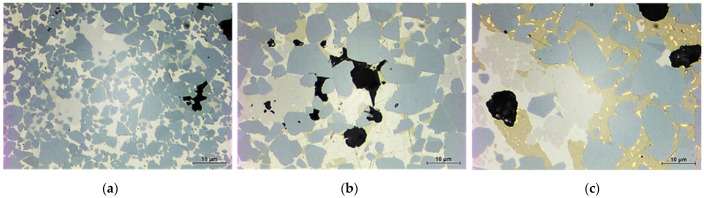
SEM micrographs of cemented carbides with different HEA binders (CoCrCuFeNi, Al_0.5_CoCrCuFeNi and Al_2_CoCrCuFeNi) after vacuum sintering for 2 h at 1500 °C. (**a**) CoCrCuFeNi; (**b**) Al_0.5_CoCrCuFeNi; (**c**) Al_2_CoCrCuFeNi. Reprinted with permission from authors of [30]. Copyright 2019 Elsevier.

Mueller-Grunz et al. substituted the usual Co-based binder using high-entropy alloys (HEAs) [30]. They used the conventional equimolar CoCrCuFeNi HEA and two different HEAs with aluminum additions, namely Al_0.5_CoCrCuFeNi and Al_2_CoCrCuFeNi [61]. The micrographs of these composites sintered in a vacuum for 2 h at 1500 °C is shown in Figure 12. It is easy to see that the shape of WC grains (they are grey in color in Figure 12) surrounded by HEAs is different from that of the conventional WC-Co composites. The WC grains surrounded by HEAs are rounded and their facets are a lot less flat than those shown in [120,121]. It appears that the WC grains surrounded by the Al_0.5_CoCrCuFeNi HEA binder are slightly more faceted than those surrounded by the equimolar CoCrCuFeNi HEA binder and Al_2_CoCrCuFeNi HEA binder with aluminum (see Figure 12). This result means that the faceting–roughening transition takes place at the WC/binder interfaces if one substitutes the simple cobalt-based binder using the HEA-based binders, presumably as a result of the suppression of recrystallization of the fine-grain WC fraction. The rounding of the WC/binder interfaces can further affect the crack initiation and propagation. This process can be especially important for cemented carbides subjected to high impact loads. Such WC change in the shape of carbide grains can, thus, deteriorate or improve the mechanical properties of WC-based cemented carbides.

**Figure 13:**
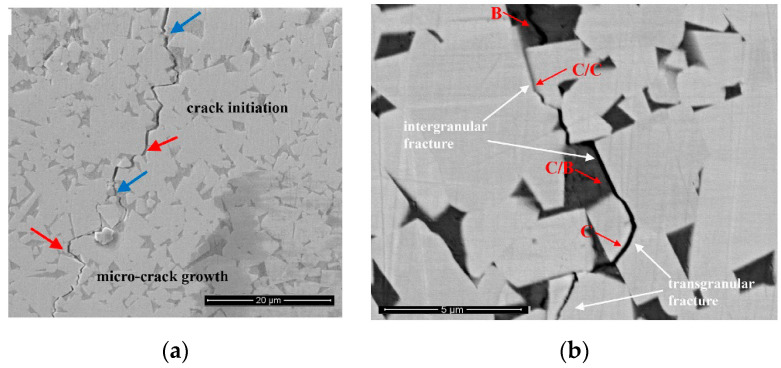
Crack growth path of WC-5Co-4Ni-1Fe (wt.%) alloy. (**a**) Pre-crack zone. Blue arrows mark intergranular fracture. Red arrows mark transgranular fracture. (**b**) Enlarged view of crack. C marks transgranular fracture, C/B and C/C mark intergranular fracture; B marks fracture through the binder. Reprinted with permission from authors of [122]. Copyright 2021 Elsevier.

The work of Qian et al. gives a good example of how the modification of WC grains’ shape can influence the mechanical properties of WC-based composite [122]. Qian et al. used vacuum sintering, followed by hot isostatic pressing (Sinter-HIP), to manufacture the WC-CoNiFe and WC-Co composites with different WC grain sizes [122]. The fracture toughness of WC-CoNiFe composite was higher than that of WC-Co and was related with the parameters of the hardmetal microstructure (Figure 13). The same was also true for the fatigue crack growth (FCG). It is clearly visible in Figure 13b how the crack propagation meets an area with a higher binder content. In this case, the FCG mode changes gradually from a transgranular (C) to an intergranular fracture (C/B and C/C) and, finally, to the fracture through the binder (B). The ductile ligament bridging and crack deflection increase the fracture toughness. The conditions required for this increase are an increase in the mean free path of the binder phase (λ_Co_) and/or a decrease in the contiguity of the carbide phase. The main toughening mechanism for WC grains is the crack deflection (Figure 13a). The shape of WC grains allows strong crack deflection due to the characteristics of the WC grains. These grains have a close-packed hexagonal structure and only one slip family {1010} <1123> in the unit cell. Consequently, they possess only four independent slip systems in this slip family. As a result, during the crack propagation, if it meets the WC grain, it is much easier to destroy the grain boundary and not the WC grain itself because it is impossible that the slip surface and direction are the same in both grains. In this way, the intergranular crack propagation is dominant over the transgranular crack propagation. In turn, the crack propagation between WC grains would strongly depend on their shape. As we can see in Figure 13, crack paths following larger WC grains are much more tortuous. They frequently have deflections or branches. Therefore, the FCG can change from a cleavage-like brittle mode to a ductile dimple mode [122]. That outcome explains is why the fatigue sensitivity of WC-Co composites is related to their fracture toughness [122].

**Figure 14:**
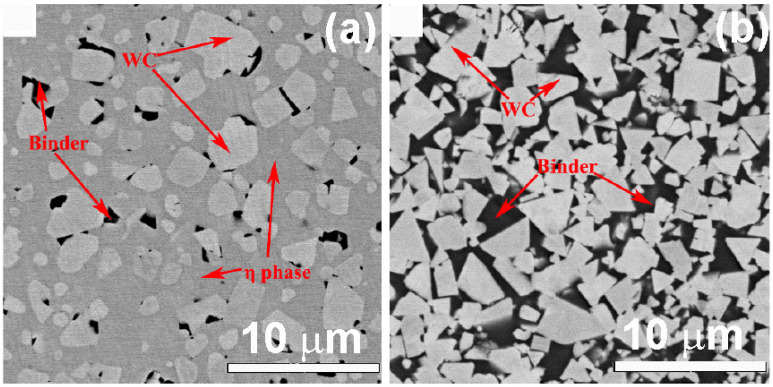
SEM micrographs showing WC crystallites surrounded by η-phase (**a**) and CoNiFeCrC-based binder (**b**). Reprinted with permission from author of [123]. Copyright 2022 Elsevier.

Qian et al. prepared the WC-10 wt.% Co-7 wt.% Ni-2 wt.% Fe-1 wt.%Cr cemented carbides with different carbon contents (4.5, 4.7 and 4.9 wt.% C) using the sintering in vacuum, followed by hot isostatic pressing (Sinter-HIP) [123]. The microstructure of these composites had the clusters of rounded WC crystallites, which were completely surrounded by η-phase (Figure 14a). The facets of rounded WC grains were less flat than the WC grains surrounded by the CoNiFeCrC binder (Figure 14b). Through increasing carbon content, the η-phase aggregated in large clusters. This coarsening of the η-phase lead the hardness to decrease and TRS to increase (see Table 1). The change in the WC grains’ shape could be at least partially responsible for this effect.

Minor shape changes among WC grains were also observed due to the substitution of cobalt binder in WC-based cemented carbides using various HEAs, such as CoNiFeCr [124,125], CoCuFeNi [126] and FeCoCrNiAl [127].

## 7. Summary of a Conducted Literature Survey and Definition of the Future Work

The above analysis shows that WC-based cemented carbides, also known as hardmetals, have great potential for further development. In particular, it outlines such a non-trivial property as the shape of individual WC grains. In the classical WC-based cemented carbides with cobalt binder, the WC grains have a truncated trigonal prism shape with very flat side surfaces (Section 1). In turn, these flat side surfaces form very sharp edges or ripples. However, various factors can change this ideal shape of WC grains. Firstly, the modification of fabrication parameters of conventional WC-Co cemented carbides can also change the shape of WC grains (Section 2). For example, many WC grains in the WC-12 wt.% Co cemented carbide fabricated via selective electron beam melting SEBM had rounded corners. Moreover, after additional sintering at 1400 °C, the rounded corners of WC crystallites were still present. The changes in the shape WC grains were also observed through the preparation of conventional WC-Co cemented carbides using laser powder bed fusion, hot isostatic pressing and spark plasma sintering, as well as through varying WC grain size in WC-Co cemented carbides with the same cobalt content along with carbon and cobalt content.

The alloying of conventional cobalt binder using various metals is another way to modify the shape of WC grains (Section 3). For example, the conventional cobalt binder was alloyed with Al, Ni and W in such a way that the Ni-to-Al atomic ratio varied (it was 0, 1.25, 2.5 and 3.75) and the Al-to-W atomic ratio was constant at 1.1 [21]. As a result, the new facets appeared in the equilibrium shape, and the corners of WC crystallites became more and more rounded with increasing nickel content. The addition of ruthenium to the cobalt binder modified the shape of WC grains [22,23]. They became slightly rounded. This effect was correlated through obtaining the maximal fracture toughness and hardness. The addition of vanadium increased hardness and decreased fracture toughness [27,33]. Vanadium strongly segregated at the WC/WC grain boundaries and WC/Co interphase boundaries. TEM studies witnessed that this segregation was facet-dependent.

The alloying of cobalt binder using nitrides, borides, carbides, silicides, oxides is another way to improve the properties of WC-based cemented carbides and change the shape of WC-Co interfaces (Section 4). In [28], three different quaternary solid-solution powders (Ti, M)(C_1−x_, N_x_) were produced with various nitrogen contents. The nitrogen content influenced the shape of WC crystallites. At *x* = 0.15, the WC grains became slightly rounded. Through an increase in the nitrogen content towards *x* = 0.20 and 0.26, the sharp corners appeared again. In turn, the transverse rupture strength and hardness followed this non-monotonous behavior of interface faceting. The WC-17 wt.% Co-based cemented carbides were manufactured via selective laser melting and reinforced with hexagonal boron nitride h-BN (10, 5 and 3 vol.%). The alloying with h-BN drastically changed the shape of WC crystallites. Instead of truncated prisms, they had a thin flat platelet shape. This transition was especially pronounced in the samples with 5 vol.% of h-BN. Such platelets contained only a few basal planes and decreased the number of stacking faults interfering with crack propagation. The samples with h-BN had better mechanical properties than the undoped WC-17 wt.% Co-based cemented carbides.

The cobalt binder could be totally substituted with other binders, including high entropy alloys (Section 5 and Section 6). The most radical modification of the shape of WC grains took place when the cobalt binder is substituted with Al–Si–10Mg alloy [64]. In this case, the WC grains had a spherical shape, which was completely different from truncated trigonal prisms in conventional cemented carbides with the Co binder. The obtained material had high Vickers hardness, good compression strength and reasonable abrasion wear resistance. The cemented carbide with the cobalt binder substituted using the 45Cr–18Ni alloy had good corrosion and oxidation resistance, as well as optimal dimensional stability [128]. Therefore, they could be applied as mould materials working at high temperatures. Such moulds require the high corrosion, wear and oxidation resistance. In these composites, not only did the edges of the WC crystallites become rounded, but about half of WC/binder interphase boundaries were also curved. The substitution of the conventional Co-based binder using high-entropy alloys (equimolar CoCrCuFeNi HEA and two different HEAs with aluminum additions Al_0.5_CoCrCuFeNi and Al_2_CoCrCuFeNi) also strongly changed the shape of WC grains [61]. They became rounded and, therefore, affected the crack initiation and propagation. Therefore, the fatigue crack growth could change from a cleavage-like brittle mode to a ductile dimple mode [25]. That result explains why the fatigue sensitivity of WC-Co composites is related to their fracture toughness.

Thus, various correlations between the shape of WC grains and mechanical properties of WC-Co cemented carbides were found in the above literature survey. Roughly speaking, the WC-Co cemented carbides with round (or less faceted) WC grains possessed better mechanical properties. The faceting of WC grains can be influenced through various methods of alloying and sintering. Therefore, this approach, which is known as grain boundary engineering, can be successfully applied to tailor the microstructure and, thus, improve the properties of WC-Co cemented carbides.

## 8. Conclusions

The WC-based cemented carbides possess the potential for further development. In particular, development can be performed with purposeful change in shape of individual WC grains. Usually, the grains have a truncated trigonal prism shape with flat facets and very sharp edges or ripples. This ideal shape of WC grains can be obtained in different ways. Useful strategies include the adjustment of production parameters of conventional WC-Co cemented carbides; alloying of conventional cobalt binder using various metals; alloying of cobalt binder using nitrides, borides, carbides, silicides, oxides; and the substitution of cobalt with other binders, including high entropy alloys. Instead of conventional truncated trigonal prisms, it is possible to obtain WC grains that have a thin flat plate shape. The sharp edges can become rounded through use of suitable compositions and production parameters, while the flat surfaces can become curved. In extreme cases, the WC crystallites can transform into spheres. Such changes can modify the ways for dislocation glide as well as the initiation and propagation of cracks. As a result, various mechanical properties can also be changed. In particular, one can see that an increase in the fracture toughness and hardness of cemented carbides correlates with a transition from a faceted to rounded shape for WC grains. Therefore, the investigation of the correlations between the shape of WC grains and various properties of WC-based cemented carbides is promising with respect to their further development.

## Figures and Tables

**Table 1:** Vickers hardness, fracture toughness and strength of WC-based cemented carbides with various binders.

Alloys’ Compositions and Sintering Temperatures, °C	Hardness, Vickers Units	Fracture Toughness, MPa·m^1/2^	Compression Strength and Transverse Rupture Strength, MPa
			Compression strength
WC-10 wt.% Co, 1500 °C [24]	1910	8.10	3595
WC-12Co after SEBM [14]	1929		1770
WC-12Co, SEBM + 1400 °C [14]	1686		1839
WC-45Cr-18Ni, binder jetting + 1350 °C [25]	>1200		>2200
WC-8Co [26] at 600 °C			1270
WC-8Co-0.8TaC [26] at 600 °C			1300
WC-8Co [26] at 800 °C			950
WC-8Co-0.8TaC [26] at 800 °C			1080
WC-Co [26] at 1000 °C			800
WC-8Co-0.8TaC [26] at 1000 °C			810
WC-12Co-*x*VC with *x* = 0 [27]	1415	12.31	
WC-12Co-*x*VC with *x* = 2 [27]	1613	12.11	
WC-12Co-*x*VC with *x* = 4 [27]	1934	12.19	
WC-12Co-*x*VC with *x* = 6 [27]	2124	11.26	
WC-12Co-*x*VC with *x* = 8 [27]	2059	10.61	
			Transverse rupture strength
(Ti, M)(C_1−x_, N_x_) (M = Ta, Nb, W, *x* = 0) [28]	1448	12.4	3780
(Ti, M)(C_1−x_, N_x_) (M = Ta, Nb, W, *x* = 0.15) [28]	1550	14.1	3320
(Ti, M)(C_1−x_, N_x_) (M = Ta, Nb, W, *x* = 0.20) [28]	1560	14.2	3430
(Ti, M)(C_1−x_, N_x_) (M = Ta, Nb, W, *x* = 0.26) [28]	1530	14.5	3500
WC-17Co-0 hBN [29]	1300	6.3	
WC-17Co-3 hBN [29]	1700	6.4	
WC-17Co-5 hBN [29]	1200	6.5	
WC-17Co-10 hBN [29]	1000	3.8	
WC-10 wt.% Co-0 wt.% Ru [22]	1200	16.5	2780
WC-10 wt.% Co-1 wt.% Ru [22]	1310	16.0	2850
WC-10 wt.% Co-2 wt.% Ru [22]	1300	17.2	2550
WC-10 wt.% Co-3 wt.% Ru [22]	1310	18.2	2200
WC-10 wt.% Co-4 wt.% Ru [22]	1330	24.2	2350
WC-10 wt.% Co-5 wt.% Ru [22]	1320	21.0	2500
WC-10 wt.% Co-6 wt.% Ru [22]	1320	20.0	2350
WC-10Co7Ni2Fe1Cr4.5C (wt.%) [30]	1080	-	1800
WC-10Co7Ni2Fe1Cr4.7C (wt.%) [30]	1000	-	1700
WC-10Co7Ni2Fe1Cr4.9C (wt.%) [30]	950	-	3450

## Data Availability

Data are contained within the article.
